# Setting the Stage for an App-Based Caregiver-Mediated Intervention for Autism: Findings from A Stakeholder Consultation

**DOI:** 10.1007/s10803-025-06890-0

**Published:** 2025-06-04

**Authors:** Monica Vanoncini, Martina Cattaneo, Gloria Falcone, Mariachiara Fioravanti, Giulia de Leonardis, Laura Zampini, Erica Salomone

**Affiliations:** Department of Psychology, University of Milano-Bicocca, Piazza dell’Ateneo Nuovo 1, U6 Building - Room 4077 (4th floor), 20126 Milano, Italy

**Keywords:** Autism intervention, Parent training, Stakeholder consultation, Mobile application, Thematic analysis

## Abstract

**Purpose:**

The use of mobile technology to support clinical practice (mHealth) has the potential to support access to caregiver-mediated interventions for autistic children, improving accessibility, scalability, and cost-effectiveness. However, rarely stakeholders’ perspectives have been integrated in the development of mHealth tools. This study aims to investigate stakeholders’ needs, attitudes, and expectations regarding a to-be-developed mHealth caregiver-mediated intervention based on the WHO Caregiver Skills Training program.

**Methods:**

We conducted focus groups and interviews with 13 caregivers and 14 clinicians.

**Results:**

Thematic analysis identified four main themes: “Usage scenarios”, “Purposes”, “Threats to app success”, and “Strategies for app success”. Stakeholders emphasized the need for an app that could support caregivers at different stages and roles, regardless of previous exposure to in-person CST. Stakeholders expected an app that could integrate psychoeducation, support home practice, and promote psychological wellbeing. Perceived threats to the app’s effectiveness pertained to the self-directed delivery, in absence of a therapist; while strategies proposed to counter such threats included incorporating engaging content, gamification and customization features, and overall ease of use of the app.

**Conclusion:**

Findings are discussed in relation to the development of a prototype based on caregivers’ needs and recommendations from expert clinicians. Future directions involve conducting formal usability testing of the initial prototype and evaluating the app’s acceptability and effectiveness.

Parent-mediated interventions for autism are promising methods to empower parents to support children in developing essential skills through naturalistic, developmental behavioral strategies (Schreibman et al., 2015) and have the potential to address the heterogeneity that characterizes autism, families, cultures, and available community resources through individualized approaches (Lord et al., 2022). The challenge lies in accessing these interventions, as many families face barriers such as travel to clinics, financial costs, and time constraints (Sapiets et al., 2021), and often lack support outside of their own resources (Lord et al., 2022), both in low- and middle-income (Reichow et al., 2013) and high-income countries (Salomone et al., 2016; Smith et al., 2020).

In response to this challenge, telehealth services, also known as eHealth, have gained popularity (Hall et al., 2016). Self-directed telehealth approaches operate without direct therapist involvement, using various communication technologies to further reduce costs and resources required for delivering interventions. This approach allows parents to participate at their convenience– during early mornings, evenings, weekends, or holidays– instead of at scheduled appointments (Ingersoll & Berger, 2015). Moreover, caregivers can access evidence-based interventions while awaiting therapist support rather than relying solely on information gathered online. A recent systematic review of telemedicine found that eHealth interventions are comparable in effectiveness to traditional “in-person” treatments and yield promising outcomes in relation to facilitating access to intervention and promoting parental empowerment and fidelity of use of intervention strategies (Micai et al., 2024). Self-directed programs are particularly beneficial when parents are fully committed, with commitment linked to treatment-related factors (i.e., expectation, acceptability, satisfaction) rather than sociodemographic variables or computer fluency (Ingersoll et al., 2023). Despite advancements, challenges remain in tailoring interventions for children, accommodating parents’ time constraints, and ensuring their familiarity with intervention delivery (Glenn et al., 2023).

Interventions supported by mobile devices, such as smartphones and tablets (i.e., mHealth; WHO, 2011) represent a further step up in making self-directed interventions personalized and feasible, as mobile applications are designed with a focus on ease of use, accessibility, portability, and the ability to meet the specific needs of caregivers of autistic children. In addition, mHealth may include features to enhance engagement in programs, an aspect playing a key role in self-directed programs (Ingersoll et al., 2023), such as gamification (i.e., the incorporation of game playing features, such as playful design elements) or virtual reality. Thus, this technology holds promise for bridging the gap in access to essential interventions for all autistic individuals, as well as their families, teachers, therapists, clinicians, and researchers. A recently proposed taxonomy of mHealth apps identified the following categories: apps supporting autistic individuals directly; apps for parental support; teacher-training apps; educational apps; apps for data collection (Liu et al., 2023).

Despite such wide range of existing tools, mHealth applications available to assist parents of autistic children present several shortcomings related to lack evidence of usability, feasibility, and efficacy (Bharat et al., 2023). For these interventions to be effective, it is essential to develop them as community-based mHealth tools (McCurdie et al., 2012; Walton et al., 2024), i.e., considering the perspectives of parents and healthcare providers from the outset. Moreover, as professionals and parents may have differing perspectives regarding goals and definitions of meaningful outcomes when dealing with children with disabilities (Leadbitter et al., 2018; McConachie et al., 2015), it is essential to develop parent-mediated interventions in keeping with a parent-carer agenda for these to be impactful. Previous research has indeed used stakeholder consultation in their formative process to guide the design of applications so that they can effectively meet the needs and perspectives of parents and healthcare providers. Specifically, with a systematic literature review regarding mHealth caregiver-mediated interventions for children with developmental disorders (Salomone et al., 2025) we identified twelve studies which included a stakeholder consultation as part of the app development cycle (Table 1). Nonetheless, despite these efforts, several common methodological limitations affect the quality of the available evidence. A notable limitation concerns the type of stakeholders being consulted. That is, some studies consulted only families (Alnaghaimshi et al., 2020; Doan et al., 2020; Premanandan et al., 2023), while others addressed only professionals (Khanahmadi et al., 2023; Reis et al., 2021; Shminan et al., 2020; Winoto et al., 2017); only five out of 12 studies incorporated input from both types of stakeholders. Furthermore, only a few studies addressed a specific condition (e.g., exclusively autism) or a particular age group of care recipients. In fact, eight studies did not specify the age of care recipients, merely reporting that the app would target children. Another concern is that, except for one study (Shminan et al., 2020), all proposed apps were not developed on evidence-based interventions, raising questions about their usefulness and effectiveness, as well as safety. Moreover, in four cases, stakeholder consultation occurred only after a prototype had been fully developed, limiting the inclusion of stakeholders’ perspectives and therefore potentially jeopardizing the relevance of the final product. Finally, the methodological issues prevalent in many of these studies– such as small sample sizes, recruitment bias, and lack of robust qualitative analysis techniques– further exacerbate these concerns and significantly restrict the conclusions that can be drawn from the findings. Taken together, these factors highlight significant gaps in current literature. Closing these gaps is critical to developing successful and impactful mHealth interventions for children with autism. This involves understanding caregivers and professionals and actively involving them in requirements definition, design, and iterative evaluation (Liu et al., 2023). Stakeholder consultation during the initial stages of concept generation and ideation is indeed crucial for investigating user needs and effectively translating them into functional requirements and design guidelines.

The goal of this study was therefore to explore stakeholders’ needs, attitudes, and expectations regarding a to-be-developed mobile application to deliver a caregiver-mediated intervention based on the World Health Organization’s Caregiver Skills Training (CST), an evidence-informed caregiver-mediated intervention originally consisting of nine in-person group sessions and three home visits (Salomone et al., 2019; WHO, 2022). Specifically, we aimed to employ qualitative methods to collect input from a wide range of stakeholders, including professionals knowledgeable about CST and target end-users of the mobile application (parents), both with and without prior exposure to CST delivered in-person, with the ultimate aim of collaboratively designing an adaptation of CST for mHealth delivery.

**Table 1:** Overview of previous studies of caregiver-mediated mHealth interventions for neurodevelopmental disorders including a stakeholder consultation

Reference	Participants	Mobile app	Condition of care recipients	Age group of care recipients	Based on evidence-based intervention?	Before (0) or after (1) a prototype?	Data analysis
Premanandan et al., 2023	Caregivers (*N* = 13; of which fathers: *n* = 2)	General e-coaching app	Diabetes, ASD, ADHD, mental development delay, Glanzmann thrombasthenia, bone issues due to aging, schizophrenia and hemiplegia, vestibular vertigo.	0–90 years	No	0	Thematic
Alnaghaimshi et al., 2020	Caregivers (*N* = 9)	Autismworld	ASD	Children	No	0	Thematic
Shin et al., 2020	Parents (*N* = 3); teachers (*N* = 3)	TalkingBoogie	Developmental disabilities dependent on AAC	6–15 years	No	0	Iterative clustering
Hwang et al., 2014	Speech and Language Pathologists (*N* = 8); parents (*N* = 13; of which fathers: *n* = 1)	TalkBetter	Language delay, ASD, pervasive developmental disorder	3–7 years	No	0	Iterative clustering
Khanahmadi et al., 2023	Occupational therapists (*N* = 15)	Sensory diet	ADHD	Children	No	0	Questionnaire
Reis et al., 2021	Occupational therapists (*N* = 4)	Regul-A	ASD	3–6 years	No	0	Unspecified
Sawyer et al., 2022	Mothers (*N* = 7); Speech and Language Pathologists (*N* = 4), teachers (*N* = 3), program administrator (*N* = 1)	Parents Plus	Developmental Language Disorder	Young children	No	1	Unspecified
Shminan et al., 2020	Expert in the Special Education field (*N* = 2)	AutiTEACCH	ASD	Children	Yes	1	Unspecified
Sonne et al., 2016	Psychologists (*N* = 3), child psychiatrists (*N* = 2), medical doctors and researchers (*N* = 3); caregivers (*N* = 2)	MOBERO	ADHD	Children	No	0 (with professionals); 1 (with families)	Unspecified
Winoto et al., 2017	Teachers (unspecified sample size)	Word-Learning Mobile Game	ASD	Children	No	1	Unspecified
Shminan et al., 2017	Speech therapists and parents (unspecified sample size)	AutiPECS	ASD	Children	No	0	Unspecified
Doan et al., 2020	Children with ADHD and their caregivers (*N* = 24 dyads)	CoolCraig	ADHD	Children	No	0	Open and axial coding

## Methods

### Participants

Participants were clinicians and parents, recruited with a purposive sampling approach through clinical centers of the Italian National Health Service. Clinicians were recruited from a pool of 20 healthcare professionals from nine Regions (in the North, Center and South of Italy) who had attended a training to qualify as CST Master Trainers delivered by the research team as a part of a joint initiative with the Italian National Institute of Health (Italian NIH; Istituto Superiore di Sanità, ISS). Parents were identified from the list of patients in the respective catchment areas of each service, aiming to recruit both individuals with previous experience of receiving the CST, as well as families without such experience. The sample size was determined to ensure a balance between too few participants, which might have limited discussions, and too many participants, which might have constrained individual input (Gill et al., 2008).

The final sample consisted of 12 healthcare female professionals with experience in delivering the CST (6 psychologists and 4 neuro-and-psychomotor developmental therapists, 1 speech and language therapist, 1 psychiatric rehabilitation therapist), and 20 caregivers (13 with previous exposure to in-person CST, and 7 without; 14 mothers and 6 fathers) caring for autistic children between 3 and 10 years old (11 males). Demographic information was available for 15 caregivers. Of these, 11 were of Italian nationality and 4 were of other nationalities highly represented in the general local population (Moldova, Spain, China, and Morocco); mean age was 39.4 years, (*SD* = 5.56). Caregiver reported annual family income was lower than the national average for 7 families, comparable for 2, and higher for 5; their educational backgrounds included secondary school (*n* = 1), high school (*n* = 5), bachelor’s degree (*n* = 4), and master’s degrees (*n* = 5).

### Procedure

The study was conducted in line with the ethical principles of the Declaration of Helsinki and the Convention on Human Rights and Biomedicine (Oviedo Convention). Ethical approval was granted by the local commission for minimal-risk studies of the Department of Psychology of the University of Milan-Bicocca (# RM-2023-654). Written informed consent was obtained from all participants. Participants were invited to attend focus groups or individual interviews, based on their availability. All focus groups (*n* = 3) and interviews (*n* = 20) were conducted online and followed similar topic guides, covering attitudes, needs, and expectations regarding a mobile application based on CST intervention strategies. Prompts included the potential relevance, acceptability, and feasibility of the mobile application; suggestions for adapting the CST into a mHealth tool, and expectations about potential difficulties and constraints. All meetings (lasting on average 90 minutes) were recorded and transcribed verbatim, excising any identifying information from the transcripts.

### Data Analysis

An inductive thematic analysis was conducted with NVivo (version 14) to identify recurring themes (Braun & Clarke, 2006). First, two coders familiarized themselves with the data through multiple readings of transcripts, and annotation of initial ideas. Second, relevant extracts were collated into descriptive codes generated independently by the two coders. Then, after a process of comparison, the coders identified a single set of coded data. Third, the analysis focused on organizing the individual codes into potential themes and subthemes, considering the relationship between the coded data and the different levels of themes. Coding conflicts were resolved by consulting an experienced third coder. Fourth, themes were reviewed in relation to the entire dataset, refining them to ensure consistency and relevance to the research question. Fifth, themes were named to adequately represent the collected codes, ensuring no overlap and a clear hierarchy of meaning within the data. Finally, themes and subthemes were narratively described and represented with a thematic map.

## Results

**Fig. 1:**
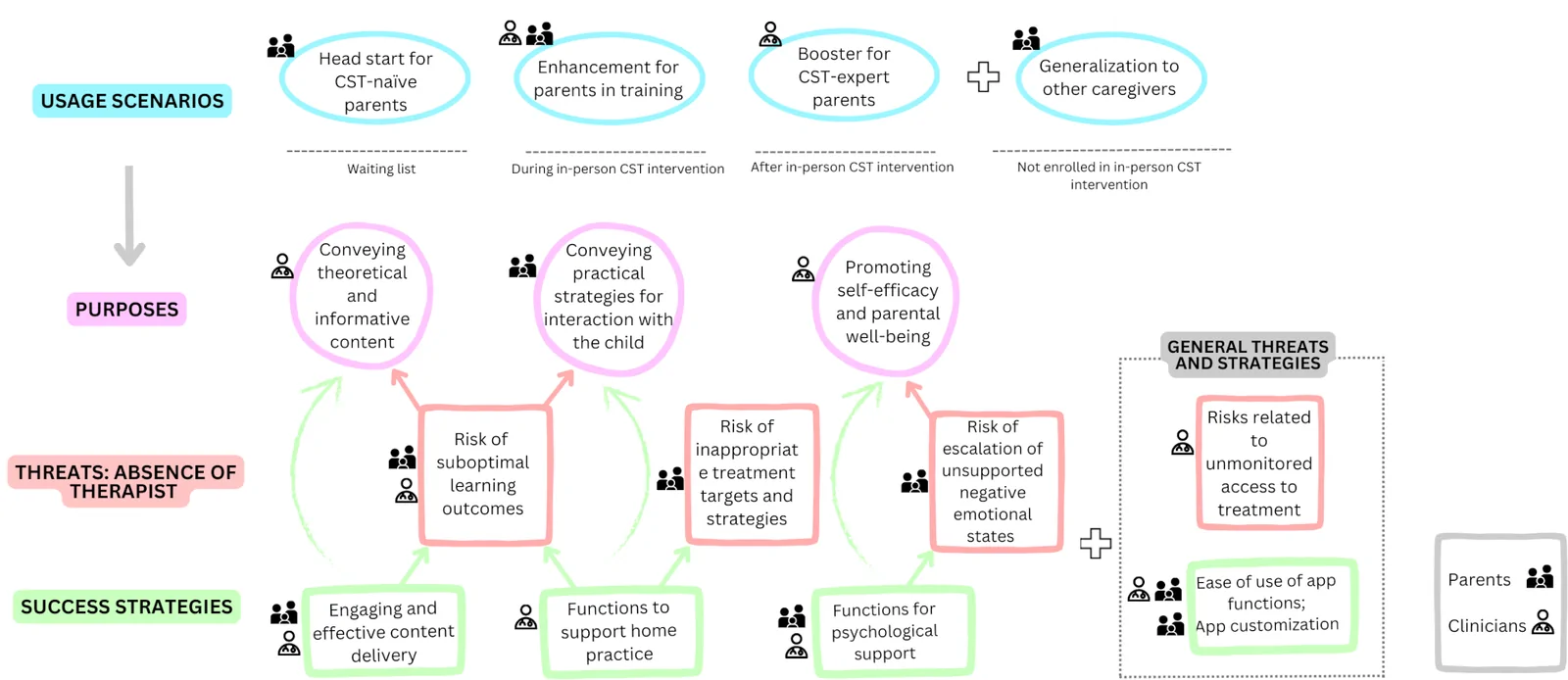
Key themes and subthemes Note. The icons of parents and clinicians placed next to each sub-theme represent the type of stakeholder that most expressed the sub-theme. When both icons are present, the subtheme was equally represented in both stakeholder groups

The analysis identified four main themes, “Usage scenarios”, “Purposes”, “Threats to success”, and “Success strategies”, each with its own subthemes. Each major theme is presented in the following paragraphs and illustrated in Fig. 1. All identified themes and sub-themes were relevant and applicable for both stakeholder groups, though certain themes held greater significance for one group compared to the other (as detailed below and represented in the Figure).

### Usage Scenarios

Overall, stakeholders expressed the view that the app should be tailored to support caregivers throughout four critical stages of their journey. Initially, it could be a valuable resource for parents who have yet to receive the in-person CST, “*so as not to be left in the lurch*” (P2; FG 18/05) (subtheme “Head start for CST-naïve parents”). With this regard, clinicians expressed reservations about widespread app distribution, advocating for its selective use among local service users. Clinicians and parents also indicated that the app could serve as a supportive tool during the delivery of the in-person CST, offering easy access to reviews and new topics to enrich the learning experience. Additionally, it could help parents who need to pause their participation for various reasons in tracking and maintaining their progress. After completing the CST sessions, the app could serve as a valuable addition, providing ongoing resources for further development. As mentioned by clinicians: *“Parents at the end of the intervention often ask ‘What is next? It was so good*,* so meaningful*,* but how can we go on?”* (C2; FG 13/07) (subtheme “Booster for CST-expert parents”). Finally, parents expressed interest in features that promote knowledge sharing among teachers and other family members, fostering a collaborative approach that strengthens the overall support network for their children (subtheme “Generalization to other caregivers”). On this topic, clinicians stressed the importance of attending the in-person CST first in order to prevent the possibility of the app potentially inappropriately replacing their role as professionals, and to ensure parents effectively understand the content. One clinician believed that a technological tool could facilitate information sharing within the family more easily and effectively than the booklets (participant guides) that are distributed to parents in the in-person CST. Taking it together, the app should be designed to support caregivers at various stages and in different roles. It should fill gaps for parents seeking reliable information while waiting for in-person services, provide resources for parents in training, serve as a booster for those who have already completed the in-person training, and also support other caregivers in the child’s life. Rather than replacing existing resources, the app could add value to existing services, providing support to caregivers in multiple ways: from essential support for treatment-naïve caregivers to more complex guidance for caregivers with prior exposure to CST, to providing the opportunity of skills generalization across contexts by facilitating information sharing among all caregivers in a child’s life. Finally, the app should be available to all caregivers, including those who for various reasons cannot participate in in-person interventions. Therefore, content should be accessible, and guide parents in a step-by-step fashion, leading them toward increasingly complex goals through self-paced delivery.

### Purposes

Parents and clinicians envisioned the app as a tool with a threefold aim: to improve caregivers’ knowledge and information, to promote practical skills for interaction with children, and to support their self-efficacy and wellbeing.

To achieve the first goal, as expressed mainly by clinicians but also by most parents, the app should incorporate essential psychoeducational content. Both clinicians and parents sought a user-friendly app with accurate, science-informed content as an alternative to paper manuals: “*Only a function of synthesis*,* of putting together the things said during the previous parent training and recalling the key messages*,* the strategies*” (C1, FG 13/07). Caregivers also requested comprehensive guides on bureaucratic matters and resources related to autism services, including professional contacts, accredited centers, and local parent associations. Moreover, as expressed mostly by parents, the app should blend theory with practice, providing engaging activities and strategies to tackle difficulties. Parents sought practical advice and information on how to deal with critical situations, such as “*strategies that can be used on a practical level in daily life*” (P1; I 29/05). In addition, a view– mainly expressed by clinicians– was that increased parental involvement could, in turn, enhance caregivers’ ability to accurately observe their child’s skills and set appropriate learning goals. Clinicians also emphasized the need for the app to support caregivers’ wellbeing, because “*if the parent is not well*,* then the child is not well either*” (C1; I 29/06). Caregivers proposed that fostering self-efficacy involves encouraging constructive engagement with their children while managing daily demands, for example, providing reminders for regular activities.

### Threats

Both groups of stakeholders identified several challenges that could threaten the success of the application, mostly stemming from the intervention being completely self-guided, without therapist assistance. As mentioned by mainly clinicians, not only such lack of guidance could cause significant misunderstandings in relation to key psychoeducational contents and intervention strategies– especially among immigrant caregivers with limited fluency in Italian– but the impossibility of monitoring attendance might cause parents to skip sessions, ultimately compromising the program integrity and contributing to higher dropout rates. Both stakeholder groups noted that, without a therapist, parents may find it difficult to set appropriate therapeutic goals tailored to their child’s interests and strengths. Clinicians expressed concerns that parents might overestimate their ability to manage their child’s needs, potentially leading to ineffective interventions. Some caregivers who attended the in-person CST training echoed this view, indicating a need for professional support in addition to the contents included in the app, “*a mobile app won’t cut it*” (P1_FG 18/05). As highlighted by some parents and clinicians, the absence of the sensitive feedback and psychological support that parents would receive during an in-person intervention may intensify feelings of isolation and frustration among parents, further affecting their engagement in the program as parents could easily feel discouraged by challenges. Another risk mentioned is that parents might develop unrealistic expectations about their child’s progress without the opportunity of discussing this with a therapist, which could in turn lead to feelings of disappointment if such expectations were not met. Lastly, clinicians expressed concern that mHealth technology might discourage attendance to in-person interventions.

### Success Strategies

Stakeholders proposed several strategies that they believed could address the challenges arising from the lack of therapist support and help reach the app’s goals. Both clinicians and caregivers emphasized the need to incorporate essential features to promote optimal user experience, including engaging content through short videos, visual aids, plain language, and multilingual options, as the app “*should be a light thing where I feel free to move around*” (P1; I 30/05). They also supported the modular organization of the CST, unlimited access to materials for parents who have attended the in-person sessions, and the inclusion of self-assessment quizzes at the end of each session. In addition, caregivers expressed the need for features for home practice, such as progress monitoring, an activity tracker, and an optional recording function for performance review. Clinicians and parents highlighted the importance of app functionalities for progress monitoring and activity tracking “*which can be motivating for them*” (C1; FG 4/07). Personalized feedback could guide suitable activities and achievable goals. Furthermore, both types of stakeholders proposed several psychological support strategies for parents, including building a support network (as highlighted by caregivers), and sending motivational notifications (as emphasized by clinicians). Clinicians also suggested retaining the breathing technique included in the in-person CST and, in general, including a dedicated wellbeing area that should be clearly marked and easily accessible to encourage caregivers to prioritize their own wellbeing.

Finally, two macro-level strategies were highlighted: ease of use and customization. Caregivers emphasized that “*simplicity should be the key*” (P2; FG 28/06), recommending a content search engine for quick topic access via keywords. Mostly parents emphasized the need for tailored feedback, to cater for the heterogeneity of autistic children, and customizable notification settings. Users should be able to add their personal activity ideas alongside the provided examples. The app could allow parents to create profiles with their child’s name, photos, and characteristics for targeted activity suggestions. Notably, customization should include data protection measures, such as password access.

## Discussion

The present study aimed to establish a robust framework for designing a mHealth tool based on a caregiver-mediated intervention. We investigated stakeholders’ needs, attitudes, and expectations regarding a to-be-developed mobile application based on CST by conducting consultations prior to the development of a prototype.

Our thematic analysis revealed four key themes reflecting these needs: “usage scenarios”, which focused on potential users and the critical stages at which the app should support caregivers; “purposes”, which envisioned the app as a tool to improve caregivers’ theoretical knowledge and practical skills, increasing their self-efficacy and wellbeing; “threats”, which concerned the various challenges that could arise especially in the absence of a therapist’s assistance; and “success strategies”, which emphasized essential features and several strategies that stakeholders believe can solve the threats. Overall, the identified themes and subthemes describe a cohesive picture including both caregivers’ and clinicians’ experiences and attitudes, but it is relevant to note that some aspects were not perceived as equally relevant in the two groups, reflecting their different perspectives of their roles in relation to the child (see Fig. 1).

On the one hand, caregivers particularly emphasized the need for the app to serve as a valuable resource for CST-naïve parents seeking guidance immediately following a diagnosis of ASD, consistent with previous studies. Similarly, Premanandan and colleagues (2023) highlighted the importance of accessible information and support for immigrant caregivers to reduce their feelings of isolation and overcome challenges such as long waiting times. Additionally, in line with previous research, families reported the need for accurate and reliable psychoeducational content, information about available services in their communities and for connections among families to share experiences (Alnaghaimshi et al., 2020). Furthermore, as previously reported (Premanandan et al., 2023), our findings show how caregivers would like to pass on the theoretical and practical aspects of the intervention to other caregivers, such as teachers or other relatives. This could represent a true advantage of the to-be-designed app, which could be potentially shared with others involved in the care of the child and could support the creation of a support network among clinicians. On the other hand, mainly clinicians advocated for the app’s use as a supplemental tool immediately following the in-person CST intervention, as a “booster” to support continued use of intervention strategies.

With respect to the goals of the to-be-designed application, in line with previous studies (Premanandan et al., 2023), clinicians emphasized the importance of incorporating theoretical content, and, drawing on their clinical experience, they recommended that the app encourages parents to focus on their personal growth and wellbeing. Parents instead stressed the importance of receiving practical strategies for interacting with their child in the context of everyday life. They also appreciated the opportunity that an app would provide to be able to rely on such support on any day, at any given time. Integrating such complementary perspectives is essential to create a balanced app that provides both practical and clinically relevant support.

Finally, both parents and clinicians clearly identified potential risks (“Threats”) associated with the app, primarily related to the nature of the self-directed intervention, i.e. a self-paced tool without therapist support. This has raised concerns among clinicians and caregivers about the possibility of suboptimal learning outcomes as well as the escalation of unsupported negative emotional states. Specifically, clinicians expressed apprehension regarding the use of the app, voicing fears of providing unmonitored access to treatment and the potential of being replaced by the app. Nevertheless, stakeholders (mainly caregivers) identified several potential solutions (“Success Strategies”) to overcome such challenges and ensure the intervention’s success. Their insights are particularly helpful as they allow the co-development of a mHealth tool that balances ease of use with the delivery of effective content, both of which have been shown in previous studies to contribute to positive outcomes (Bharat et al., 2023).

### Strengths and Limitations

This study has a number of methodological strengths. To the authors’ knowledge, this is the first study of stakeholder consultation for the development of an application delivering contents of a parent-mediated intervention which examined the perspective of multiple stakeholders (professionals, mothers, fathers), focused specifically on ASD, and employing rigorous inductive thematic analysis methods, gathering insights from an adequately sized sample on a range of topic guides, from needs assessment to desirable features. We opted for involving stakeholders at the outset of the application development process, to incorporate from the start diverse perspectives that could enhance the design and functionality of the application, ultimately leading to the design of a more supportive and effective treatment experience. This approach aligns with previous studies (e.g., Shminan et al., 2020; Winoto et al., 2017), but differs from others, such as Sawyer and colleagues (2022), who gathered feedback only after the development of prototype. Previous studies often failed to report stakeholder consultation results clearly (e.g., Khanahmadi et al., 2023; Reis et al., 2021) and others lacked specific information on methods for data analysis (e.g., Sonne et al., 2016), further limiting the interpretation of findings (see also Salomone et al., 2025).

Despite the significant advancements introduced by the current study, certain limitations cannot be overlooked. First of all, in order to facilitate caregiver participation, we employed both focus groups and interviews; this may have introduced variability in the amount of detail provided that could have impacted the overall findings. Additionally, while a strength of the study lies in the heterogeneity of the sample, which included participants from several different Regions of Italy, the nature of the recruitment process might have led to a selection bias, as all participants were drawn from clinical centres of excellence that had been selected by the Italian NIH to participate in a CST Master Trainer training course. This may have resulted in a sample mainly consisting of highly motivated professionals and parents, potentially limiting the generalizability of the results to a wider population. Finally, while the involvement of stakeholders from the outset can be an asset in order to incorporate their views from the beginning, it should also be considered that in absence of an existing prototype stakeholders may have struggled with visualizing how their needs could potentially be met by an application or may have put forward unrealistic expectations. In our case, while some of the suggested features could easily be developed, others, such as the embedded social network for caregivers, would pose complex technological and ethical issues.

Our qualitative approach allowed us to gain insights into the perspectives of our stakeholders, marking a crucial first step in the development of a new mHealth caregiver-mediated intervention based on the CST. By involving stakeholders in the development of the app, we laid the groundwork for designing a comprehensive system with functionalities that have the potential to meet users’ needs, thereby enhancing the likelihood of producing a relevant, well-designed, and effective tool. Based on the insights gathered from this stakeholder consultation, we have subsequently proceeded to developing a prototype for the application, i.e. a preliminary interactive version of the app which includes design and functions, but not a working code. Findings of the prototype testing with target users which indicated good usability and acceptability to parents are reported elsewhere (de Leonardis et al.,^2025^). The next steps involve the evaluation of acceptability, feasibility and effectiveness of the fully developed app with pilot testing with a randomized controlled design. With such an approach we aim to follow all the necessary– yet to date to the authors’ knowledge never previously completed– phases of a comprehensive development cycle for a mHealth tool delivering a caregiver-mediated intervention.

## Data Availability

In accordance with our commitment to transparency and collaborative research, data will be made available upon request.
